# High-CBD Extract (CBD-X) Downregulates Cytokine Storm Systemically and Locally in Inflamed Lungs

**DOI:** 10.3389/fimmu.2022.875546

**Published:** 2022-05-16

**Authors:** Miran Aswad, Haya Hamza, Antonina Pechkovsky, Anastasiia Zikrach, Tania Popov, Yaniv Zohar, Eduardo Shahar, Igal Louria-Hayon

**Affiliations:** ^1^ Medical Cannabis Research and Innovation Center, Rambam Health Care Campus, Haifa, Israel; ^2^ Pathology Department, Rambam Health Care Campus, Haifa, Israel; ^3^ Clinical Immunology Unit, Rambam Health Care Campus, Haifa, Israel; ^4^ Clinical Research Institute at Rambam (CRIR), Rambam Health Care Campus, Haifa, Israel

**Keywords:** cannabis, corona, cytokine storm and inflammation, CBD - cannabidiol, COVID - 19, lung inflammation

## Abstract

Cytokine storm refers to the dysregulated production of inflammatory mediators leading to hyperinflammation. They are often detrimental, and worsen the severity of COVID-19 and other infectious or inflammatory diseases. Cannabinoids are known to have anti-inflammatory effects but their possible therapeutic value on cytokine storms has not been fully elucidated. *In vivo* and *ex vivo* studies were carried out to investigate the effects of high-THC and high-CBD extracts on cytokine production in immune cells. Significant differences between the extracts were observed. Subsequent experiments focusing on a specific high CBD extract (CBD-X) showed significant reductions in pro-inflammatory cytokines in human-derived PBMCs, neutrophils and T cells. *In vivo* mouse studies, using a systemically inflamed mouse model, showed reductions in pro-inflammatory cytokines TNFα and IL-1β and a concurrent increase in the anti-inflammatory cytokine IL-10 in response to CBD-X extract treatment. Lung inflammation, as in severe COVID-19 disease, is characterized by increased T-cell homing to the lungs. Our investigation revealed that CBD-X extract impaired T-cell migration induced by the chemoattractant SDF1. In addition, the phosphorylation levels of T cell receptor (TCR) signaling proteins Lck and Zap70 were significantly reduced, demonstrating an inhibitory effect on the early events downstream to TCR activation. In a lung inflamed mouse model, we observed a reduction in leukocytes including neutrophil migration to the lungs and decreased levels of IL-1β, MCP-1, IL-6 and TNFα, in response to the administration of the high-CBD extract. The results presented in this work offer that certain high-CBD extract has a high potential in the management of pathological conditions, in which the secretion of cytokines is dysregulated, as it is in severe COVID-19 disease or other infectious or inflammatory diseases.

## Introduction

The term cytokine storm was first coined in 1993 by Ferrara et al. describing graft-versus-host disease (1). It is a potentially fatal immune disease involving multiple organs (2). Immune cells are highly activated, resulting in the production and release of an overload of inflammatory cytokines and chemokines leading to hyperinflammation. A cytokine storm is not only characterized by abnormally high levels of circulating cytokines, but is accompanied by the appearance of thrombi, severe lymphopenia and immune cell infiltration into several organs (3), caused by an imbalance in the regulation of the immune system (4).

A cytokine storm is always detrimental. Patients that develop it show a rapid onset of fever, cytopenias, coagulopathy, elevated transaminases, hyperferritinemia and multiple organ dysfunctions (5). It can be accompanied by macrophage-activation-syndrome (MAS), which is characterized by the production of IL-1β, IL-6, IL-18 and interferon-γ (IFNγ) (6).

The likelihood of the development of cytokine storm in rheumatologic diseases such as idiopathic arthritis or lupus erythematosus as well as in multiple sclerosis (7), pancreatitis (8) or multiple organ dysfunction syndrome (9, 10) is well-known. In the field of infectious diseases, cytokine storms were first reported in the early 2000 for several viruses among them influenza virus (11), avian influenza H5N1 virus (12), cytomegalovirus (13), Epstein-Barr virus (14), variola virus (15) and severe acute respiratory syndrome-coronavirus (SARS-CoV) and SARS-CoV2 (16, 17). A cytokine storm in Coronavirus-disease-19 (COVID-19) is accompanied by the rapid release of pro-inflammatory cytokines IL-6 and TNF-α (18). The parallel activation of the NF-κB pathway and the subsequent activation of the STAT3-pathway result in a signal-amplifying cascade releasing other pro-inflammatory cytokines from immune and non-immune cells involving the angiotensin II type 1 receptor, which can induce the release of TNF-α, among other cytokines (19). IL-6 has an important role in this process because its trans-activation *via* gp130 results in pleiotropic effects on innate as well as acquired immune cells, leading to enhanced cytokine release resulting in a cytokine storm (20).

The cytokine storm is the result of an impairment of the sophisticated and complex balance between pro- and anti-inflammatory cytokines. Different classes of cannabinoids, including the psychoactive phytocannabinoid tetrahydrocannabinoid (THC) and the non-psychoactive phytocannabinoids such as cannabidiol (CBD) show anti-inflammatory action (21, 22). The endocannabinoid system plays a key role in the immune response of the innate and the adaptive immune systems. It modulates the migration of hematopoietic stem and progenitor cells (23) and regulates the cell trafficking of mature immune and effector cells, such as lymphocytes, macrophages, neutrophils and dendritic cells (24). It was reported that pure CBD and THC extracts could attenuate the proliferation of activated lymphocytes and the secretion of pro-inflammatory cytokines, thereby increasing the secretion of the anti-inflammatory IL-10 (25). In addition, THC could induce immunosuppression in B cells (26). However, the mechanistic basis for the effects of cannabis on systemic hyperinflammation and the cytokine storm, and locally on lung inflammation, is not yet fully understood.

In this study, we have examined the anti-inflammatory properties of six cannabis strains, three of them with high-THC and three with high-CBD content. We revealed that one of the high-CBD-extracts, termed CBD-X, had an enhanced anti-inflammatory capacity compared to the other CBD strains. We will show that CBD-X extract reduced the levels of the pro-inflammatory cytokines TNF-α, IL-6 and IFN-γ while increasing the anti-inflammatory cytokine IL-10 in primary human derived neutrophils and T cells. In a murine model displaying systemic and local lung inflammation, it was shown that the administration of high-CBD extract downregulated the migration of leukocytes to the site of infection and the development of cytokine storm and leukocyte migration, both systemically, and locally in the lung tissues.

## Materials and Reagents

ELISA kits for mouse TNF-α, IL-1β, IL-10, IL-6 and MCP-1 or for human TNF-α, IFN-γ, IL-6 and IL-8 or Human TruStain FcX™ (Fc Receptor Blocking Solution), anti-human CD3 (mouse, IgG2a, κ, clone OKT3), anti-human CD28 (mouse, IgG1, κ, clone CD28.2), human IL-2, PE anti-human CD3 antibody (mouse, IgG1, κ, clone UCHT1), APC anti-human CD4, (mouse, IgG2b, κ, clone A17070D) and Pacific blue™ anti-human CD8 (mouse, IgG1, κ, clone SK1), TruStain FcX™ (rat anti-mouse CD16/32, IgG2a, Λ, a) and APC anti-mouse CD45 (rat, IgG2b, κ, clone 30/F11) were obtained from BioLegend, CA, USA.

The media DMEM and RPMI 1640 Medium with L-Glutamine were obtained from Biological Industries, Beit Haemek, Israel and the medium X-VIVO 15 with gentamicin and phenol red was obtained from Lonza (Switzerland).

Human Neutrophil Isolation Kit, Human CD4^+^ T -Cell Isolation Kit and Lymphoprep-Density gradient medium were obtained from STEMCELL Technologies, Vancouver, Canada. Millicell Hanging Cell Culture Inserts, PET 5 µm, 24-well were purchased from Merck, NJ, USA.

Lipopolysaccharide (LPS) was obtained from Santa Cruz (CA, USA). Human SDF1 was obtained from PeproTech, London, UK. Fetal bovine serum (FBS), glutamine and penicillin and 100 U/ml streptomycin were obtained from Biological Industries, Beit Haemek, Israel.

Mouse anti-phospho STAT5 and rabbit anti STAT5, rabbit anti-phospho Lck and mouse anti Lck, rabbit anti-phospho Zap70 and mouse anti–Zap70 as well as mouse anti β-Actin were obtained from Cell Signaling Technology (MA, USA). 2x Laemmli Sample Buffer was purchased from Biorad and β-mercaptoethanol from Merck Millipore.

### Cannabis Extracts

Cannabis extracts were kindly provided by Raphael Pharmaceutical Inc Nevada, USA. The strains were cultivated and grown by Way Of Life Cannabis (WOLC), Israel. Cannabis extracts were either high-THC or high-CBD extracts. To distinguish the different extracts, they were termed THC-A, THC-B, THC-C, CBD-X, CBD-Y, CBD-Z.

The cannabinoids percentage in a concentration of 1 µg/ml is as follow:

THC-A contains: 71.7% THC, 0% CBD, 0% CBN, 0% CBG
THC-B contains: 53% THC, 0.1% CBD, 0.4% CBN, 0.4% CBG
THC-C contains: 59% THC, 0.1% CBD, 0.2% CBN, 1% CBG
CBD-X contains: 35% CBD, 0.3% THC, 0% CBN, 0.3% CBG
CBD-Y contains: 62% CBD, 8.5% THC, 0.1% CBN, 0% CBG
CBD-Z contains: 75% CBD, 3.6% THC, 0% CBN, 0% CBG

### Human Peripheral Blood Samples

Human peripheral blood samples were obtained from the Israeli National Biobank for Research (MIDGAM) at Rambam Health Care Campus. The experiments were authorized by the Helsinki Committee at Rambam health Care Campus (Authorization No. 0442-20 RMB).

### Mice

C57BL/6 mice were obtained at the age of 8 to 10 weeks from Envigo, Israel. All mice were housed at a barrier/free and specific pathogen/free animal facility at the Pre-Clinical Research Authority, Technion-Israel Institute of Technology in Haifa, Israel. All experiments were performed according to the regulations of the Inspection Committee on the Constitution of the Animal Experimentation of the Technion-Israel Institute of Technology in Haifa, Israel from which authorization for performing animal studies was approved (Authorization No. IL-0950820). Experiments conformed to the regulations in the Prevention of Cruelty to Animals Law (Experiments on Animals) 5754-1994 and the Prevention of Cruelty to Animals Rules (Experiments on Animals) 5761-2001 Correct as of December 1, 2005.

### Cell Culture

The RAW 264.7 mouse macrophages were cultured in DMEM supplemented with 10% heat-inactivated FBS, 3 mM glutamine and 100 U/ml penicillin and 100 U/ml streptomycin at 37°C under a humidified atmosphere of 5% CO2. Subcultures are prepared by scraping cells from floor of dishes every two days and diluting to 1 x 10^6 cells/20 ml. Medium renewal is 2 to 3 times per week.

In all experiments, cells were allowed to acclimate overnight before treatments.

### Screening of the Effects of Cannabis Extracts

The mouse macrophages cell line RAW264.7 cells were seeded at a concentration of 1 x 10^6^ cells/ml into a 96-well plate in full DMEM medium and incubated at 37°C and 5% CO_2_ overnight. Cells were washed and cannabinoid extracts from different strains, termed were added at a concentration of 0.7 µg/ml and incubated for three hours. Cells were washed and incubated with 0.5 µg/ml lipopolysaccharide (LPS) in full DMEM medium. 16 hours later, cells were centrifuged, supernatants were collected, and the concentration of secreted IL-6 was determined using the IL-6 ELISA kit (BioLegend).

### Cell Viability Measurement

Mouse macrophages (RAW 264.7 cells) were incubated in DMEM overnight and treated with different cannabinoid extracts at concentrations of 1, 2, 3 or 4 µg/ml of the extract. Incubation lasted for three hours, then cells were washed, and medium was added to the cells with 10% Alamar Blue solution. As a negative control, Alamar Blue was added to the medium without cells. The cells were further incubated for another four hours at 37°C. The absorbance of the test and control wells was read at 560 nm and 590 nm with a standard spectrophotometer.

### Isolation of Immune Cells From Blood

Peripheral blood samples of healthy volunteers were collected in EDTA-containing tubes. Neutrophils were isolated from blood samples by negative magnetic selection with the EasySep Direct Human Neutrophil Isolation Kit (STEMCELL Technologies) according to the manufacturer’s instructions. Briefly, Isolation Cocktail (50µl/ml) and RapidSpheres (50µl/ml) were added to a whole blood sample tube for 5 minutes. The sample tube was topped up with recommended medium and inserted into the magnet for 10 minutes. Then, sample was transferred to a new tube and RapidSpheres (50µl/ml) were added for an additional 5-minute incubation. The sample with the RapidSpheres was inserted into the magnet for 5 minutes and sample was transferred to a third tube. The last step was repeated to obtain a final clear fraction.

Peripheral blood mononuclear cells (PBMCs) were isolated by Lymphoprep-Density gradient medium (STEMCELL Technologies) according to manufacturer’s instructions. Briefly, the diluted blood (in 2% FBS in PBS) was layered on the Lymphoprep. Tubes were centrifuged at 800 x g for 20 minutes. The plasma layer was then discarded, and the layer of mononuclear cells (MNC) was isolated.

CD4^+^ T cells were isolated by EasySep Direct Human CD4^+^ T-cell Isolation Kit according to the manufacturer’s instructions (STEMCELL Technologies). In brief, isolation cocktail (50 µl/ml) and RapidSpheres (50 µl/ml) were added to the blood samples for 5 minutes. Samples were topped up with recommended medium. The sample tubes were inserted into the magnet for a further 5 minutes. The samples were transferred to a new tube. RapidSpheres (50 µl/ml) were once more added to the samples. These were inserted into the magnet for another 5 minutes. The final step was repeated to get a clear fraction.

### Cell Culture and Treatment of the Cells With Cannabis Extracts

RAW 264.7 mouse macrophages were cultured in DMEM supplemented with 10% heat-inactivated FBS, 3 mM glutamine, and 100 U/ml penicillin and 100 U/ml streptomycin at 37°C in a humidified atmosphere of 5% CO_2_. In all experiments, cells were allowed to acclimate overnight before treatments.

1 x 10^6^ cells/ml isolated neutrophils, PBMC derived T cells or CD4^+^ T cells were treated with 2 µg/ml cannabis extract or DMSO as a control for two hours. Neutrophils were cultured in X-VIVO 15 and PBMC derived T cells or CD4^+^ T cells in RPMI 1640. After incubation, CBD-X treated cells and control cells were centrifuged and activated or left untreated. Neutrophils were activated with 100 ng/ml LPS in X-VIVO 15 medium overnight. PBMC derived T cells or CD4^+^ T cells were activated by seeding them on a plate coated with 2.5 µg/ml anti-human CD3 and 2.5 µg/ml anti-CD28 in RPMI 1640 medium to which 125 units/ml IL-2 were added for three days. Treated cells were centrifuged, supernatants were collected and levels of TNF-α, IL-6 and IFN-γ were detected by enzyme linked immunosorbent assay (ELISA) according to manufacturer’s instructions (BioLegend).

### Western Blot Analysis for Human Isolated Cells

CD4^+^ T cells were isolated from healthy donors by negative magnetic selection with the EasySep Direct Human CD4^+^ T cells Isolation Kit. Isolated CD4^+^ T cells were activated with 5 µg/ml anti-CD3 and 5 µg/ml anti-CD28 and treated with 1 or 2 µg/ml CBD-X or DMSO as a control. After an hour, the cells were collected and pelleted by centrifugation at 18800 x g and washed twice with ice-cold PBS. The washed cell pellets were resuspended in 100 µl 2x Laemmli Sample Buffer with added β-mercaptoethanol at a ratio of 1:20 (SB+βME), incubated at 100 °C for 5 min and centrifuged at 18800 x g for 5 min; cell pellet containing debris was discarded. The protein samples were separated on a 10% SDS-PAGE gel and transferred onto nitrocellulose membrane using Trans-Blot Turbo Transfer Apparatus (Bio-Rad) following manufacturer’s instructions. The membrane was blocked with 5% BSA in Tris-buffered saline containing 0.1% Tween 20 followed by incubation overnight at 4°C with total or phospho-specific antibodies to STAT5 (1:500), Lck (1:1000), and Zap70 (1:1000) in 5% BSA in Tris-buffered saline containing 0.1% Tween 20. β-actin (1:1000) was added as loading control. Blots were washed with Tris-buffered saline containing 0.1% Tween 20 and incubated with the respective secondary antibodies conjugated with horse radish peroxidase for one hour at room temperature. Blots were washed with Tris-buffered saline containing 0.1% Tween 20. Immunoreactive proteins were detected with the Enhanced Chemiluminescence (ECL) kit (Thermo scientific). The relative density of the protein bands was scanned using Image Quant LAS4000 and analyzed by Image-J 1.8.0-172.

### Cell Migration

For the cell migration experiments, 5 µm 24-well Boyden chambers were used. 0,5×10^6^ cells/ml of activated PBMC derived T cells were seeded in the upper chamber of the 24-well Boyden chamber in medium. In the lower chamber 100 nM of the chemoattractant SDF-1 were placed. Increased concentrations of cannabis extract (1, 2 and 3 μg/ml) or DMSO as a vehicle functioning as a negative control were added to the cells. Four hours later, cells that were migrated through the pores into the lower chamber were collected and counted by flow cytometry. PBMC derived T cells were stained with Human TruStain FcX™ (Fc Receptor Blocking Solution), anti PE- CD3, APC- CD4 and anti-Pacific blue-CD8.

### Murine Model of Systemic Inflammation

C57BL/6 mice 8 to 10 weeks of age were injected intravenously with 100-150 mg/kg cannabis extract or a vehicle (5% kolliphor, 5% ethanol, 90% saline) as a control. Simultaneously, 1 mg/kg LPS or PBS as a control were injected, intraperitoneally. After 2 h, mice were anaesthetized by isoflurane, blood was drained directly from the heart, and the peritoneal contents were collected by lavage using PBS. Blood and peritoneal fluids were centrifuged twice at 18800 x g for 10 minutes in a tabletop centrifuge. Supernatants were collected and stored at 4°C overnight. TNF-α, IL-1β and IL-10 cytokines were detected by enzyme linked immunosorbent assay (ELISA).

### Murine Model of Lung Inflammation

C57BL/6 mice 8 to 10 weeks of age were injected intravenously with 100-150 mg/kg cannabis extract or a vehicle (5% kolliphor, 5% ethanol, 90% saline) as a control. Simultaneously, 1 mg/kg LPS or PBS as a control were administered intranasally to activate lung inflammation. Two hours post-LPS activation, the mice were anaesthetized with isoflurane and sacrificed. A lung bronchoalveolar lavage (BAL) was performed with PBS. Lung fluids were centrifuged, supernatants were collected and levels of TNF-α, IL-1β and MCP-1 were detected by ELISA (BioLegend). Alternatively, lung fluids were collected, as described above, 24 hours post-LPS activation, and IL-6 secretion was detected by ELISA in the supernatant. The cells in the remaining pellet after centrifugation were stained with TruStain FcX™ (anti-mouse CD16/32) antibody (BioLegend) and APC anti-mouse CD45 (BioLegend), and analyzed by flow cytometry

### Histological Analysis

Lungs were taken from sacrificed mice and fixed with 4% paraformaldehyde (PFA) for 24h, paraffin embedded, and 4µm sections were stained with hematoxylin and eosin (H.E.). Representative photos from each sample were taken.

### Statistical Analysis

For statistical analysis, the *ex vivo* experiments data were analyzed in comparison to activated treatment as a baseline. In the case of neutrophils, the significance was determined in comparison to the “LPS, DMSO” group. while in the case of PBMC-derived T cells or CD4^+^ T cells, the significance was determined in comparison to “cell activation, DMSO” group. The T cell or CD4 activation has been done by anti CD3 and anti CD28. The data were normalized to the activated control of each donor and the average of the percentage of change was calculated from (8-13) healthy donors.

In the *in vivo* experiments, data were analyzed in comparison to LPS-induces mice as a baseline named “LPS, DMSO” group. Means were calculated of at least three biologically independent experiments. When each experiment included 5 mice in each group. Data were analyzed by one-way ANOVA (Fisher’s LSD test with values p < 0.05 considered statistically significant, (*p <0.05, **p < 0.01, *** p < 0.001).

## Results

### A Specific High CBD Extract Downregulates the Secretion of Pro-Inflammatory Cytokines From Macrophage and Human Derived Neutrophil Cells

**Figure 1:**
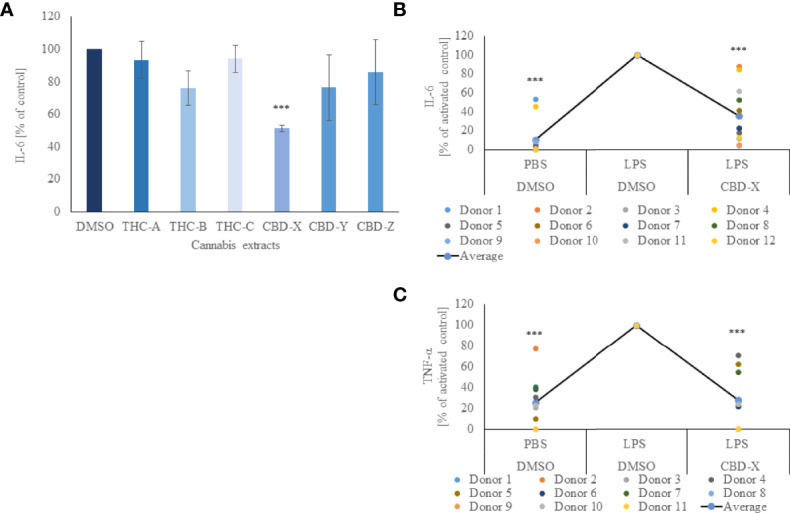
CBD-X downregulates the secretion of pro-inflammatory cytokines. **(A)** Mouse macrophages (RAW 264.7 cells) were incubated in DMEM overnight and treated with different cannabinoid extracts at equal concentrations of the respective cannabinoid. Incubation lasted for three hours. High THC-extracts were termed THC-A, THC-B, THC-C and high CBD-containing extracts were termed CBD-X, CBD-Y and CBD-Z. Medium containing cannabinoids was discarded, and cells were incubated with 0.5 µg/ml LPS overnight. IL-6 cytokine levels were detected by ELISA. Means were calculated and normalized to DMSO treated cells. Error bars represent the standard deviations of the means of three biologically independent experiments. **(B, C)** Neutrophils were isolated from the blood of healthy donors by negative magnetic selection with the EasySep Direct Human Neutrophil Isolation Kit. Isolated neutrophils were treated with 2µg/ml CBD-X or DMSO (vehicle) as a control for two hours. Treated cells were activated by 100 ng/ml LPS overnight. Levels of IL-6 **(B)** and TNF-α **(C)** were detected by ELISA. Each colored dot represents one donor. The means were calculated from healthy donors (black line) and each dot represents one case. Data were normalize in ratio to "LPS, DMSO" group and analyzed by one-way ANOVA (Fisher's LSD test with values p < 0.05 considered statistically significant, (***p < 0.001).

In order to determine the anti-inflammatory potential of the cannabis extracts, we investigated their effect on the secretion of pro-inflammatory cytokine IL-6 from mouse macrophages. To this end, the murine macrophage cell line RAW264.7 was treated with different kinds of high-THC or high-CBD extracts, termed THC-A, THC-B, THC-C, CBD-X, CBD-Y or CBD-Z, a vehicle was used as a control and IL-6 secretion was determined by ELISA. High-THC extracts slightly lowered IL-6 secretion but this reduction was not statistically significant compared to cells treated with vehicle only. High-CBD extracts CBD-Y and CBD-Z reduced IL-6 secretion slightly more than high-THC extracts, but the reduction was still not statistically significant. Interestingly, only CBD-X extract displayed a statistically significant reduction and lowered IL-6 secretion by 50% compared to vehicle control (**Figure 1A**).

To exclude the possibility that this observation was due to a cytotoxic effect of the added extract, cell viability was measured with Alamar Blue assay. Cell viability was not reduced with the addition of the cannabinoid samples, allowing us to conclude that the reduction of IL-6 secretion was due to a regulatory effect of CBD-X (**Figure S1**).

We further concluded that different cannabinoid extracts have different effects on IL-6 secretion, leading to the assumption of cannabis strain-specific effects on the regulation of inflammation. For this reason, all further experiments were conducted with CBD-X extract.

Next, we investigated whether this anti-inflammatory effect could be observed on human primary immune cells. We focused on neutrophils as they are the first line of defense against pathogens and the orchestrator of the action of other immune cells at the site of infection (27).

Thus, isolated human-derived neutrophils were pre-treated with 2 μg/ml CBD-X extract or vehicle as a control and activated with 100 ng/ml lipopolysaccharide overnight to secrete cytokines. CBD-X extract inhibited in a statistically significant manner the release of the pro-inflammatory cytokines IL-6 and TNF-α by 76% and 58% respectively compared to the control (**Figures 1B, C**). The viability of neutrophils was not altered by the applied CBD-X extract with the concentration used (**Figure S2**).

This confirms the obtained results on the murine macrophages that CBD-X extract downregulates the secretion of pro-inflammatory cytokines in immune cells displaying an anti-inflammatory effect.

### CBD-X Extract Attenuates the Secretion of Pro-Inflammatory Cytokines From PBMC-Derived T Cells or CD4^+^ T Cells

T cells were shown to play a critical role in COVID-19 disease related to lung inflammation. In fatal COVID-19 cases, the number of regulatory T cells and time-dependent escalation in activated CXCR4^+^ T cells was demonstrated (28). These phenomenon supports a model whereby lung-homing T cells activated through bystander effects contribute to immunopathology, while a non-suppressive SARS-CoV-2-specific T cell response limits pathogenesis and promotes recovery from severe COVID-19 (28).

**Figure 2:**
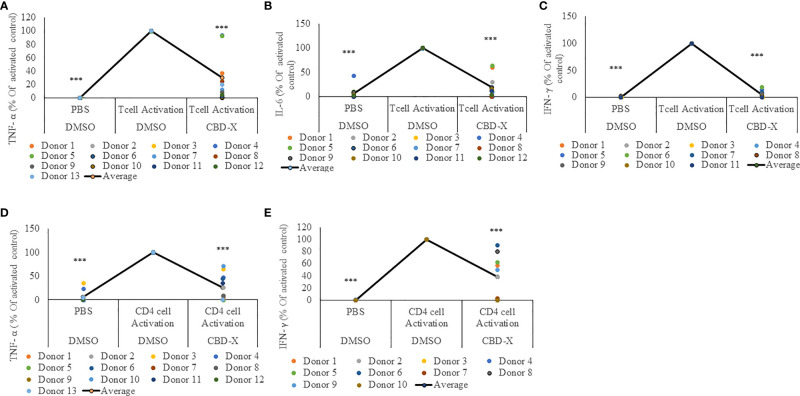
CBD-X extract attenuates the secretion of pro-inflammatory cytokines from activated human PBMC-derived T cells or CD4^+^ T cells. PBMCs were isolated from healthy donor blood and were treated with CBD-X or DMSO as a control in RPMI 1640 for two hours. Then, anti-CD28 and anti-IL-2 were added to the cells and cells were transferred to a cell culture plate coated with anti-CD3, for three days. Cells were centrifuged, supernatants were collected, and levels of pro-inflammatory cytokines TNF-α **(A, D)**, IL-6 **(B)** and IFN-γ **(C, E)** were detected by ELISA. **(A–C)** Secreted cytokines from PBMCs are shown. **(D, E)** Cytokines from CD4^+^ T cells are shown. The means were calculated from healthy donors (black line) and each dot represents one case. Data were normalized in ratio to activated cells treated with DMSO group and analyzed by one-way ANOVA (Fisher's LSD test with values p < 0.05 considered statistically significant, (***p < 0.001).

For this reason, we investigated the effect of CBD-X extract on T cells. PBMC-derived T cells from the blood of healthy human donors were activated with anti-CD3 and anti-CD28. Activated cells were treated with CBD-X extract or with vehicle as a control. The secretion of the key cytokines IL-6 and additionally of TNF-α and IFN-γ, which promotes the cytotoxic function of T cells, were examined. CBD-X significantly downregulated the secretion of the cytokines from activated T cells by 76%, 88% and 99% respectively (**Figures 2A–C**). There was no impact on T-cell viability by the cannabinoids with the concentrations used (**Figure S3**).

Recently it was demonstrated that a response from CD4^+^ T cells might be associated with COVID-19 severity (29). These findings, together with our results, prompted us to investigate the effect of CBD-X extract on CD4^+^ T cells. Human derived CD4^+^ T cells were isolated, activated and treated with CBD-X as before, and the cytokine levels of TNF-α and IFN-γ was determined. Our results show that CBD-X extract downregulated the secretion of TNF-α and IFN-γ levels by 65% and 61% respectively (**Figures 2D, E**).

We concluded that CBD-X extract has an anti-inflammatory effect on activated T cells and CD4^+^ T cells and thereby reduces the cytotoxic activity of the two investigated immune cell types.

### CBD-X Extract Modulates TCR-Signaling in Human Derived CD4^+^ T Cells

T cell receptor (TCR) activation promotes a signaling cascade and determines the cell’s destiny through the regulation of the secretion of the cytokines (30). STAT5, the signal transducer and activator of transcription protein plays a key role in T -cell proliferation *via* the T cell receptor (TCR) (31). Activation of TCR results in the activation of a signaling cascade involving Lck and Zap70 (30).

**Figure 3:**
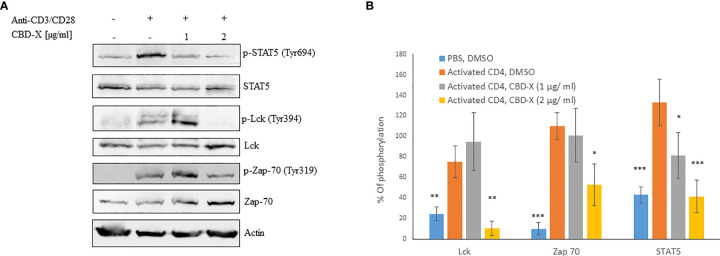
CBD-X extract modulates TCR- signaling in human derived CD4^+^ T cells. CD4^+^ T cells were isolated from healthy donors by negative magnetic selection with the EasySep Direct Human CD4^+^ T cells Isolation Kit. Isolated CD4^+^ T cells were activated with 5 µg/ml anti-CD3 and 5 µg/ml anti-CD28 and treated with 1 or 2 µg/ml CBD-X extract or DMSO as a control. After an hour, the cells were collected and lysed with 100 µl 2x SB+βME. **(A)** Cell lysates were analyzed for the presence of phosphorylated STAT5, Lck and Zap70 by western blot analysis using pY694-STAT5, total STAT5, pY394-Lck, total Lck, pY319-Zap70 and total Zap70 antibodies. **(B)** Induction of phosphorylation was quantified relative to the specific total protein expression. Error bars represent the standard deviation of the means of three different donors, and they are expressed as average ± standard deviation (SD). SD were calculated in ratio to activated CD4 (treated with Anti-CD3/CD28), and data were analyzed by one-way ANOVA (Fisher’s LSD test with values p < 0.05 considered statistically significant, (*p <0.05, **p < 0.01, ***p < 0.001).

To get insight into the mechanism of the inhibitory effect of CBD-X on CD4^+^ T -cells, we investigated whether CBD-X extract affects the signaling events of TCR activation. Therefore, human derived CD4^+^ T cells were isolated, activated by anti-CD3 and anti-CD28 antibodies, and treated with CBD-X extract. The cells were collected and analyzed for the activation of the key components of the TCR-signaling pathway by investigating the phosphorylation levels of STAT5, Lck and Zap70 by Western blot analysis. Activation of CD4^+^ T cells by anti-CD3 and anti-CD28 increased the phosphorylation levels of Lck, Zap70 and STAT5. Treatment with CBD-X extract downregulated the phosphorylation levels of Lck and Zap70 drastically more at the higher concentration of CBD-X whereas the phosphorylation levels of STAT5 were inhibited in a dose dependent manner (**Figure 3A**). Quantification of the phosphorylation status of the proteins in correlation to the total protein levels confirmed the results from the Western blots, including the statistically significant down-regulation of the phosphorylation of the tested proteins following the addition of CBD-X extract (**Figure 3B**).

From these results, we concluded that CBD-X extract interferes with the early events of TCR signaling, indicating that it is an early regulator and can inhibit pro-inflammatory events at an early stage of T-cell activation.

### CBD-X Extract Inhibits the Migration of T Cells Induced by SDF-1

**Figure 4:**
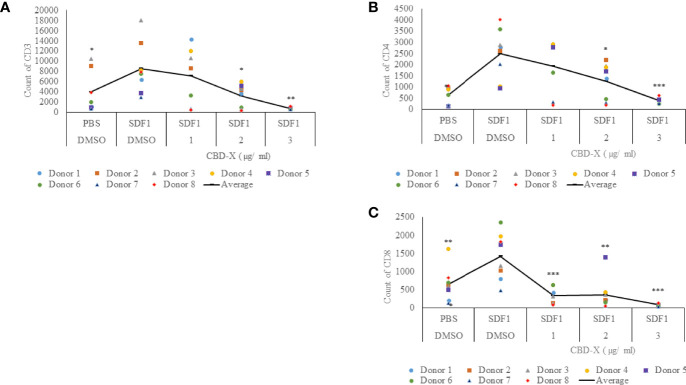
CBD-X extract inhibits the migration of T cells induced by SDF-1. PBMCs were isolated from the blood of healthy donors and leukocytes were isolated by the aid of the Lymphoprep-Density gradient medium. Leukocytes were activated by 2.5 µg/ml anti-CD3, 2.5 µg/ml anti-CD28 and 125 units/ml IL-2 for 48 hours. Activated PBMC derived T cells were seeded onto the 5 µm pore polyester membrane in the upper chamber of a 24-well Boyden chamber in RPMI 1640 medium. Human chemoattractant SDF-1 in a concentration of 100 nM was placed in the lower chamber. CBD-X extract in the concentrations of 1, 2 and 3 µg/ml or DMSO as a vehicle were added to the cells. After four hours, cells that have been migrated through the pores into the lower chamber were collected and stained with the Fc Receptor Blocking Solution Human TruStain FcX™ and anti PE- CD3, APC- CD4 and anti-Pacific blue-CD8 to distinguish T cell subtypes and subjected to flow cytometry analysis. **(A)** The number of migrated CD3^+^ T cells **(B)** of migrated CD4^+^ T cells and **(C)** of migrated CD8^+^ T cells were determined. The means were calculated from healthy donors (black line) and each dot represents one case. Data were analyzed in comparison to “SDF1, DMSO” group by and analyzed by One-way ANOVA (Fisher's LSD test with values p < 0.05 considered statistically significant, (*p < 0.05, **p < 0.01, ***p < 0.001).

Severe COVID-19 is characterized by enhanced activation of lung-homing CXCR4+ T cells, therefore, inhibiting their activity or translocation to the lungs may help reduce disease severity (28). Stromal cell-derived factor-1 (SDF1) is a ligand for the chemokine receptor CXCR4 and plays a vital role in tissue-specific migration of CXCR4^+^ T cells (32). To study the effect of CBD-X extract on chemokine-induced migration of T cells, PBMC-derived T cells were treated with 1-3 μg/ml CBD-X extract and their ability to migrate towards SDF-1 across a polyester membrane of a Boyden chamber was tested. Migrated cells were stained with anti-CD3, anti-CD4 and anti-CD8 antibodies to distinguish different subtypes of T cells by flow cytometry analysis. CBD-X extract inhibited the migration of T cells towards SDF-1 in a dose-dependent manner compared to vehicle-treated control. Migration of CD3^+^ T cells was inhibited by 10%, 54% and 86%, respectively (**Figure 4A** and **Supplementary S4**), the migration of CD4^+^ T cells by 27%, 48% and 76% (**Figure 4B** and **Supplementary S4**) and of CD8^+^ T cells by 79%, 70% and 91% (**Figure 4C** and **Supplementary S4**).

These observations suggest that CBD-X extract has the potential to interfere with the homing of T cells in the case of severe lung inflammation as is seen in COVID-19 pneumonia.

### CBD-X Extract Modulates Cytokine Secretion in a Murine Model of Systemic Inflammation

So far, our results support the notion that the high-CBD extract derived from CBD-X strain, downregulate cytokine secretion from human derived immune cells, in *ex-vivo* experiments. Next, we investigated whether cytokine secretion is also inhibited during systemic inflammation.

**Figure 5:**
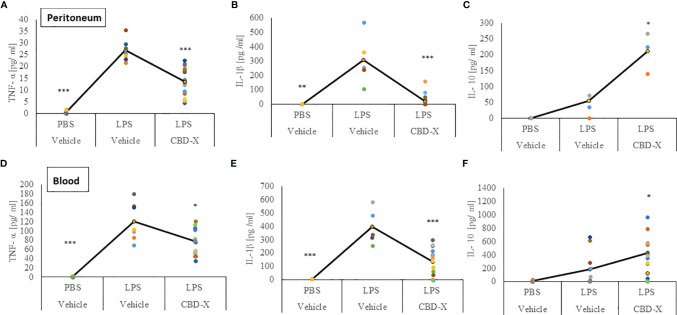
CBD-X extract promotes immune reprogramming in a murine model of systemic inflammation. C57BL/6 mice were injected intravenously with 100- 150 mg/kg CBD-X extract or vehicle as a control. Simultaneously, 1 mg/kg LPS or PBS as a control were injected intraperitoneally. After two hours, mice were euthanized. Then, blood was drained from the heart and peritoneal contents were collected using PBS through lavage. Blood was centrifuged and supernatants were collected. Levels of TNF-α, IL-1β and IL-10 from the peritoneum (upper panel, **A–C**) or blood (lower panel, **D–F**) were detected by ELISA. Each colored dot represents one mouse. Standard deviations were calculated as the means of three biologically independent experiments (black line), each experiment included five mice per group. Data were analyzed by one-way ANOVA (Fisher’s LSD test with values p < 0.05 considered statistically significant, (*p < 0.05, **p < 0.01, ***p < 0.001).

C57BL/6 mice were injected intravenously with 100-150 mg/kg CBD-X extract or vehicle as a control. Simultaneously, 1 mg/kg LPS or PBS as a control were injected intraperitoneally. Two hours later, the mice were sacrificed, their blood was drained, and peritoneal fluids were collected by PBS lavage. The secretion of pro-inflammatory cytokines TNF-α and IL-1β and the anti-inflammatory cytokine IL-10 were measured both from the blood and the peritoneum (**Figure 5**).

The increased levels of the pro-inflammatory cytokines TNF-α and IL-1β in the peritoneum from LPS-treated mice were reduced by 52% and 95% by the CBD-X extract respectively (**Figures 5A, B**). In accordance with this, the levels of the anti-inflammatory cytokine IL-10 were increased by 4 folds (**Figure 5C**).

Similar measures were made in the blood. Here, CBD-X extract inhibited the increased levels of TNF-α and IL-1β from LPS-treated mice by 35% and 65% respectively (**Figures 5D, E**). Importantly, IL-10 increased twofold in LPS-treated mice that were treated with CBD-X extract (**Figure 5F**). Interestingly, results in the supplementary data show that only CBD-X extract with no LPS induction has no significant effect on leukocyte cell number nor cytokine secretion levels in the blood baseline state (**Figures S5, S6**).

Our results indicate that CBD-X extract decreased pro-inflammatory cytokines both in the peritoneum and in the blood of LPS-treated mice while simultaneously increasing the anti-inflammatory cytokine IL-10. Thus, CBD-X extract may promote immune-reprogramming and attenuate systemic inflammation.

### CBD-X Extract Modulates Cytokine Secretion in a Murine Model of Lung Inflammation

Lung inflammation results from tissue injury caused by infectious agents or traumatic events such as toxins, pollutants, irritants, or injury. Lung homeostasis is maintained by a sophisticated balance in the release of pro- and anti-inflammatory mediators in the lung tissue and by the number of infiltrated immune cells (33).

**Figure 6:**
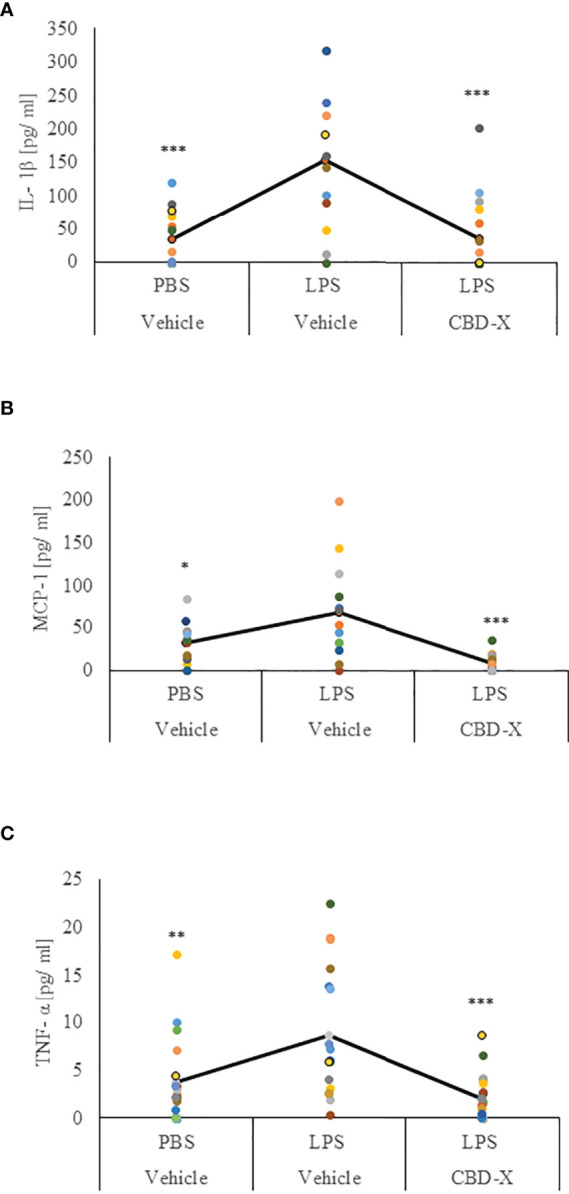
CBD-X extract promotes immune silencing in a murine model of lung inflammation. C57BL/6 mice were injected intravenously with 100- 150 mg/kg CBD-X extract or vehicle as a control. Simultaneously, 1 mg/kg LPS or PBS as a control were administered intranasally. After two hours, mice were euthanized, and lung fluids were collected using PBS through lavage. The lung fluids were centrifuged, and supernatants were collected. Levels of the pro-inflammatory IL-1β **(A)**, MCP-1 **(B)** and TNF-α **(C)** were detected by ELISA. Each colored dot represents one mouse. Standard deviations were calculated as the means of three biologically independent experiments (black line), each experiment included five mice per group. Data were analyzed by one-way ANOVA (Fisher’s LSD test with values p < 0.05 considered statistically significant, (*p <0.05, **p < 0.01, ***p < 0.001).

Our results regarding the anti-inflammatory activities of high CBD extract encouraged us to investigate whether CBD-X extract affects the levels of secreted cytokines in the lungs in LPS-treated mice. Levels of IL-1β, MCP-1 and TNF-α in the lung fluids of LPS-treated mice were inhibited with CBD-X extract by a 77%, 91% and 75%, respectively (**Figures 6A–C**).

These results indicate that CBD-X extract has an anti-inflammatory effect locally in LPS-induced lung inflammation suggesting that the CBD-X extract may contribute to the attenuation of lung inflammation.

### CBD-X Extract Inhibits Leukocyte Migration to Inflamed Lungs

Leukocyte migration precedes the migration of immune effector cells to the site of infection or injury in the lung interstitium (34).

**Figure 7:**
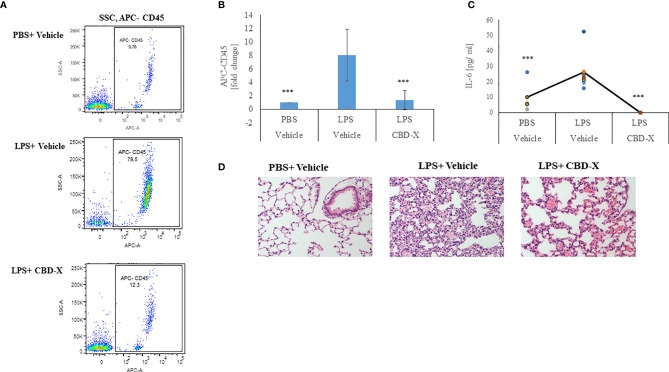
CBD-X extract inhibits migration of leukocytes to inflamed lungs LPS treated mice were injected intravenously with 150 mg/kg CBD-X extract or vehicle as a control. After 24 hours, mice were sacrificed, and lung fluids were collected. **(A)** Cells in the lung fluids were stained with TruStain FcX™ (anti-mouse CD16/32) and anti-mouse APC-CD45 for flow cytometry analysis. Vehicle-treated control cells are shown in the upper panel, LPS-treated cells in the middle panel and LPS and CBD-X treated cells in the lower panel. **(B)** Fold of change in APC-CD45 was calculated. Vehicle-treated control cells were set to 1 and LPS and LPS+CBD-X-treated cells were calculated to vehicle-treated control. Error bars represent the standard deviation of the mean of five mice/per group of three biologically independent experiments in ratio to control. **(C)** The cytokine IL-6 was detected by ELISA in the lung fluid. The means were calculated (black line) and each dot represents one mouse. Data were analyzed in comparison to “LPS, Vehicle” group by one-way ANOVA (Fisher's LSD test with values p < 0.05 considered statistically significant, (***p < 0.001). **(D)** Histological images of neutrophils infiltrated into the lungs of mice. Vehicle-treated neutrophils (left image), LPS-treated neutrophils (middle image) and LPS+CBD-X-treated neutrophils are shown. Representative images of one mice of each group of three different biologically independent experiments are shown at a magnification of X200.

Therefore, we examined whether high-CBD extract affect leukocyte migration into the lungs of LPS-treated mice. Isolated immune cells from the lung tissue were examined for the leukocyte marker CD45 by FACS analysis. Our results show that lungs from untreated mice contained 9.78% CD45 positive cells, while lungs from LPS-treated mice had a remarkable increase in the CD45 population, with up to 80% CD45 positive cells. Treatment with CBD-X extract reduced the number of CD45 positive cells to 12.3%, which is a reduction almost to baseline level (**Figure 7A**). Fold of change in CD45 population was calculated in comparison to control (**Figure 7B**). Next, we checked the levels of the pro-inflammatory cytokine IL-6 in the lung tissue, since it is a major player in migration and inflammation (35). In the lung tissue of untreated mice 5 pg/ml IL-6 was measured. When mice were treated with LPS the levels increased to 24pg/ml, which were lowered to a value below baseline to almost 0 pg/ml when CBD-X extract was administered to LPS- treated mice (**Figure 7C**). Histology images from lungs of each experimental group (Control, LPS +Vehicle and LPS+CBD-X) were analyzed 24 hours after treatments (**Figure 7D**). Focal neutrophil infiltration and congestion were noticed at the lungs of LPS treated mice, that were reduced in the mice treated with CBD-X extract (**Figure 7D**).

From these results we concluded that CBD-X inhibits leukocyte migration to damaged lungs and reduces local IL-6 secretion, thus reducing inflammation in the inflamed tissue. Over all our data support the hypothesis that CBD-X extract exhibits a potential to downregulate cytokine storm in the case of acute inflammation.

## Discussion

Cytokine storm is a detrimental hyperinflammatory disease which can be treated with anti-inflammatory medications such as anakinra that downregulates pro-inflammatory IL-1β (36). Since anti-inflammatory medications can have severe side effects and cytokine storm can be fatal despite treatment, preventing the development of hyperinflammation at an earlier stage with little or no side effects may be a better form of treatment or prevention. Here, we showed that one of the six high-THC or high-CBD extracts (termed CBD-X) presented enhanced anti-inflammatory effects. That different cannabis extracts have diverse effects was also shown earlier when the prevention of COVID-19 disease was investigated. This group has shown that different high-CBD strains had a varying potential to inhibit the entry of SARS-CoV2 into the host cell *in vitro* by modulating the ACE2 protein (37). Similarly, it is widely accepted that high-CBD has anti-inflammatory properties and can indirectly improve anti-inflammatory effects (38). In our study, we went a step further by demonstrating a direct effect of a specific high-CBD extract on inflammation by reducing anti-inflammatory cytokines secreted from immune cells but also regulating the translocation of pro-inflammatory T cells to the site of infection. The exact mode of action still needs to be elucidated but our observations hint to an involvement of early events in T -cell activation *via* the TCR signaling.

Furthermore, inhibition of migration as shown in our study was also seen in clinical studies (39) strengthening our observations.

During the inflammatory process, the secretion of cytokines mediates the communication between immune cells and their activation. This guides the defense network consisting of a plethora of immune cells, each of them with a distinct function, against invading pathogens (40). An unregulated release of pro-inflammatory cytokines as seen in severe COVID-19 disease can lead to increased mortality (41). Key regulators of the pro-inflammatory response are the pro-inflammatory cytokines IL-6, TNF-α, IFN-γ and interleukins such as IL-1β or IL-2 among others (40). We focused our study on the key pro-inflammatory cytokines IL-6, TNF-α, IFN-γ and interleukins such as IL-1β because they were shown to be elevated in severe COVID-19 patients with severe disease. Moreover, increased circulating IL-6 levels are associated with higher mortality in patients with severe COVID-19 disease (42).

Our results provide evidence that the high-CBD extract – CBD-X, is associated with a dampened signaling response to LPS-stimulation or TCR-activation *ex vivo* following CD3/CD28 stimulation and reduces the secretion of pro-inflammatory cytokines in diverse types of immune cells such as neutrophils and T cells, key regulators of the innate and adaptive immune system (43). Moreover, the activation of STAT5 proteins is a very early event that is mediated by IL-2 family cytokines (44), which is mainly produced by activated CD4^+^ T cells and less by activated CD8^+^ T cells (45). The activation of STAT5 and downstream proteins was inhibited by CBD-X extract suggesting that CBD-X has a potential to prevent severe COVID-19 disease at an already very early stage of the disease.

Furthermore, CBD-X extract downregulates the migration of CD4^+^ and CD8^+^ T cells in response to the chemoattractant SDF1 and thus may reduce the translocation of pro-inflammatory immune cells to the site of infection. This may explain the lower number of leukocytes in the inflamed lungs of LPS-treated mice administered the CBD-X extract. Most interestingly, in a systemic inflammation model, CBD-X extract promoted immune reprograming by reducing the secretion of the pro-inflammatory cytokines IL-1β and TNF-α and concurrently increasing the secretion of the anti-inflammatory cytokine IL-10. IL-10 is an anti-inflammatory cytokine that mediates the process of counteracting the pro-inflammatory immune response, an important event to prevent damage to the host tissue caused by pro-inflammatory cytokines (46). The increase of IL-10 in the blood of LPS-treated mice suggest that certain high-CBD extract, in our case CBD-X, have the potential to help resolve the immune response of innate and adaptive immunity in order to maintain the afflicted tissue in a healthy condition (46). The influence of CBD on IL-10 is not studied in detail and a clinical study on patients with multiple sclerosis did not find evidence of an influence of CBD on the increase of the anti-inflammatory cytokine IL-10 (47) but a more recent study on activated encephalitogenic T cells showed that IL-10 was increased by high-CBD extracts (48). On the other hand, the usage of cannabis as a medicinal drug revealed contradicting results in clinical studies (49), although this might be due to the diverse effects of the different cannabis strains. Thus, clinical studies are needed to confirm our results that were obtained *ex vivo* and *in vivo* in mice studies. Moreover, we conducted our experiments on cells from healthy donors. In a next step, we would investigate the influence of CBD-X extract on cells isolated from the blood of patients that were hospitalized due to an infection with SARS-CoV2. One more challenge in the field of cannabis based treatment, is the capacity to apply the cannabis extracts to the patients. It is critical that the delivery system of cannabis extracts will match the patients status. In this study we injected the extract to the mice vein or peritoneum. However for human cytokine storm patients, a delivery system should be developed according to the patient’s health condition and the target organs and cells. We believe that this strategy will make the treatment applicable and will allow an efficient cannabis based treatment to the cytokine storm patients. Nonetheless, the presented results are promising because they indicate that CBD-X extract not only regulated cytokines secretion in systemic and local inflammation, but also modified migration of immune effector cells and interfered with the downstream signaling of the activated T cell receptors. Our data point to the prospect that certain high-CBD extract has a high potential in the management of pathological conditions, in which the secretion of cytokines is dysregulated as it is in severe COVID-19 disease or other infectious or inflammatory diseases.

## Data Availability Statement

The original contributions presented in the study are included in the article/**Supplementary Material**. Further inquiries can be directed to the corresponding author.

## Ethics Statement

The studies involving human participants were reviewed and approved by Helsinki Committee, Rambam Health Care Campus, authorization no. 0442-20- RMB. The patients/participants provided their written informed consent to participate in this study. The animal study was reviewed and approved by Committee on the Constitution of the Animal Experimentation of the Technion-Israel Institute of Technology in Haifa, Israel from which authorization for performing animal studies was approved (Authorization No. IL-0950820).

## Author Contributions

IL-H conceptualized the manuscript. MA, HH, AP, and IL-H designed research. MA, HH, AP, AZ, TP, and YZ performed research. MA, HH, AP, YZ, ES, and IL-H analyzed data, MA, HH, and IL-H wrote the manuscript. All authors contributed to the article and approved the submitted version.

## Funding

This work was supported by RAMBAM MED-TECH Grant and by Raphael Pharmaceutical Inc. Cannabis and Covid-19 related Cytokine Storm Research Grant.

## Conflict of Interest

This study received funding from Raphael Pharmaceutical Inc. The funder was not involved in the study design, collection, analysis, interpretation of data, the writing of this article or decision to submit it for publication. All authors declare no other competing interests.

## Publisher’s Note

All claims expressed in this article are solely those of the authors and do not necessarily represent those of their affiliated organizations, or those of the publisher, the editors and the reviewers. Any product that may be evaluated in this article, or claim that may be made by its manufacturer, is not guaranteed or endorsed by the publisher.

## Supplementary Material

Supplementary Figure S1 Cannabinoids have no cytotoxic effect on the viability of RAW 264.7 cells. The graph represents the viability of mouse macrophages (RAW 264.7) that were treated with high THC-containing extracts, termed THC-A (dark blue line), THC-B (red line), THC-C (grey line) and high CBD-containing extracts, termed CBD-X (green line), CBD-Y (yellow line) and CBD-Z (light blue line). Standard deviations were calculated in ratio to vehicle treated cells, and data were analyzed by one-way ANOVA (Fisher’s LSD test with values p < 0.05 considered statistically significant, (*p <0.05, **p < 0.01, *** p  0.001)

Supplementary Figure S2 Cannabinoids have no cytotoxic effect on the viability of human neutrophils. The graph represents the viability of human-derived neutrophils that were treated with 1-4 µg/ml CBD-X (LPS (+)/1, LPS (+)/2, LPS (+)/3 LPS (+)/4), LPS (LPS (+)/vehicle) or vehicle only (LPS(-)/vehicle). The means were calculated from healthy donors (black line) in ratio to vehicle treated cells and each dot represents one case. Data were analyzed by one-way ANOVA (Fisher’s LSD test with values p < 0.05 considered statistically significant, (*p <0.05, **p < 0.01, *** p < 0.001).

Supplementary Figure S3 Cannabinoids have no cytotoxic effect on the viability of PBMCs. The graph represents the viability of human-derived PBMCs that were treated with 2 or 4 μg/ml CBD-X (T-cell activation/2), (T-cell activation/4), vehicle only (T-cell activation/DMSO) or left untreated (Control/-). The means were calculated from healthy donors (black line) in ratio to activated control and each dot represents one case. data were analyzed by one-way ANOVA (Fisher’s LSD test with values p < 0.05 considered statistically significant, (*p <0.05, **p < 0.01, *** p < 0.001).

Supplementary Figure S4 Migration of human PBMC-derived T cells.Shown results are representative scatter plots of the flow cytometry of migrated PBMC-derived T cells. Cells were cultivated on a pore membrane in the upper chamber of a 24-well Boyden chamber (insert) and allowed to migrate to the chemoattractant SDF1 after treatment with 1-3 µg/ml CBD-X ([(+), (+), 1 µg/ml], [(+), (+), 2 µg/ml] [(+), (+), 3 µg/ml]), vehicle ([(+), (-), (-)]) or left untreated ([(-), (-) ,(-)]). Scatter plots representing Forward scatter (FSC) against Side scatter (SSC) are shown in the upper panel, scatter plots representing Forward scatter width (FSC-W) against CD3+ T cells stained with PE (PE-CD3) in the middle panel and scatter plots representing CD8+ T cells stained with PE (PE-CD8) against APC-CD4 in the lower panel.

Supplementary Figure S5 CBD-X extract effect on leukocytes, T cells and neutrophils count in the baseline state. C57Black mice were injected with CBD-X (100 mg/Kg) or Vehicle two times a week for two months' period, intraperitoneal. Blood was drained from the tail, centrifuged and pellet was treated for 5 minutes with ACK lysing buffer (0.5 ml) to remove red blood cells. Cells were stained with TruStain FcX™ (anti-mouse CD16/32), anti-mouse APC-CD45, anti- mouse BV786-LY6G and anti-mouse FITC-CD3 for flow cytometry analysis. The gating strategy is (A) FSC vs SSC, (B) APC-CD45 vs SSC and (C-D) BV786-LY6G vs FITC-CD3. (C) Vehicle-treated control cells are shown in the upper panel and (D) CBD-X treated cells in the lower panel. (E) The means and standard deviations of leukocytes, T cells and neutrophils were calculated and data were analyzed by one-way ANOVA (Fisher’s LSD test with values p < 0.05 considered statistically significant, (*p <0.05, **p < 0.01, *** p < 0.001).

Supplementary Figure S6 CBD-X extract effect on cytokine secretion in the baseline state. C57Black C57Black mice were injected with CBD-X (100mg/Kg) or Vehicle two times a week for two months' period, intraperitoneal. Mice were sacrificed, blood was drained and levels of TNF-α, MCP-1 and IL-6 were detected by ELISA. The means and standard deviations were calculated from each group. Data were analyzed by one-way ANOVA (Fisher’s LSD test with values p < 0.05 considered statistically significant, (*p <0.05, **p < 0.01, *** p < 0.001).
