# Supplementary material for: High-CBD Extract (CBD-X) Downregulates Cytokine Storm Systemically and Locally in Inflamed Lungs
Source: Front Immunol. 2022 May 16;13:875546. doi: 10.3389/fimmu.2022.875546 (PMC9149302; doi:10.3389/fimmu.2022.875546)
Supplement: Supplementary Figure S1 — Cannabinoids have no cytotoxic effect on the viability of RAW 264.7 cells. The graph represents the viability of mouse macrophages (RAW 264.7) that were treated with high THC-containing extracts, termed THC-A (dark blue line), THC-B (red line), THC-C (grey line) and high CBD-containing extracts, termed CBD-X (green line), CBD-Y (yellow line) and CBD-Z (light blue line). Standard deviations were calculated in ratio to vehicle treated cells, and data were analyzed by one-way ANOVA (Fisher’s LSD test with values p < 0.05 considered statistically significant, (*p <0.05, **p < 0.01, *** p 0.001) [file Presentation_1.pptx]

## Slide 1
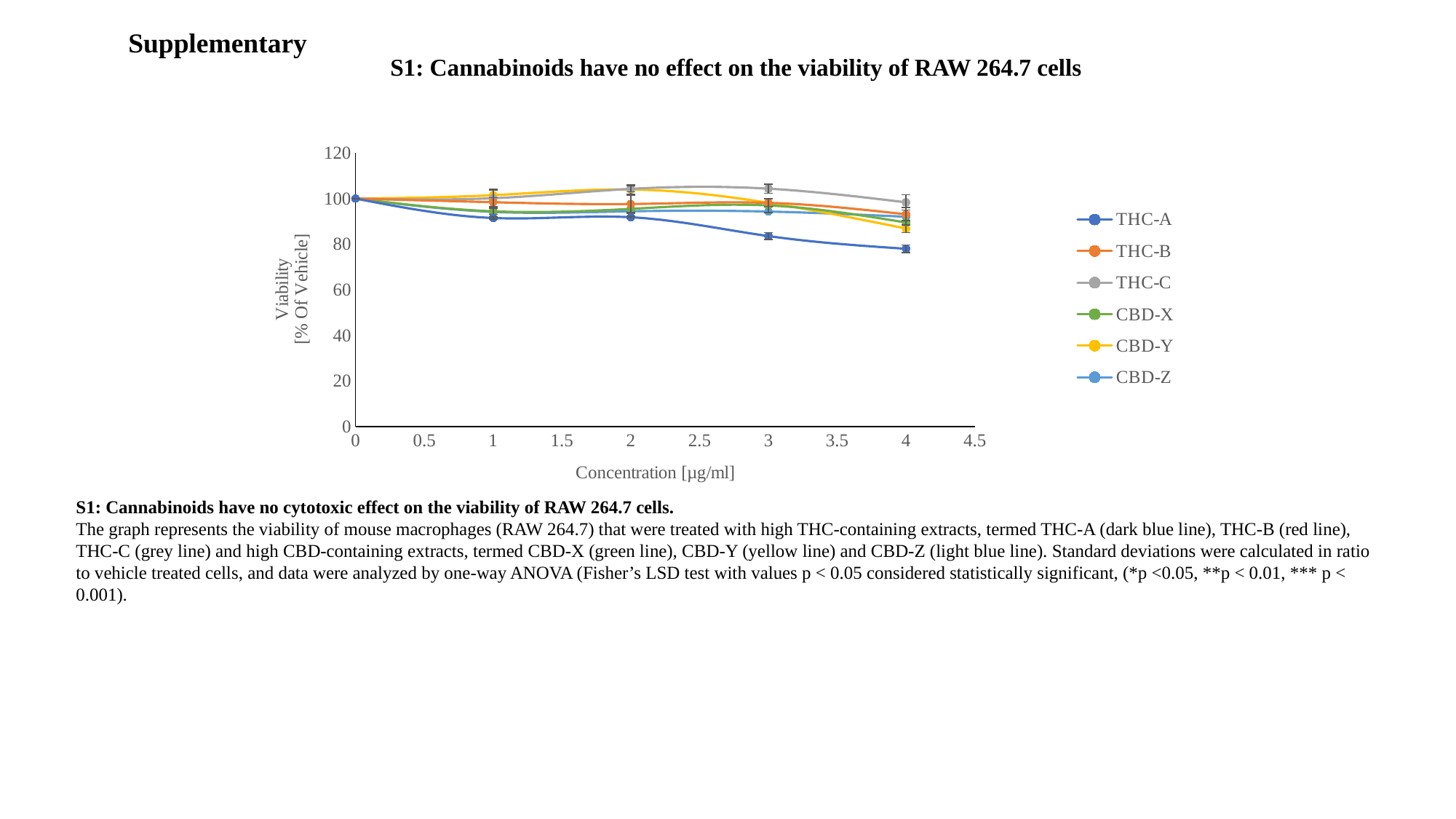

Supplementary
S1: Cannabinoids have no effect on the viability of RAW 264.7 cells
### Chart
| Category | THC-A | THC-B | THC-C | CBD-X | CBD-Y | CBD-Z |
|---|---|---|---|---|---|---|S1: Cannabinoids have no cytotoxic effect on the viability of RAW 264.7 cells.
The graph represents the viability of mouse macrophages (RAW 264.7) that were treated with high THC-containing extracts, termed THC-A (dark blue line), THC-B (red line), THC-C (grey line) and high CBD-containing extracts, termed CBD-X (green line), CBD-Y (yellow line) and CBD-Z (light blue line). Standard deviations were calculated in ratio to vehicle treated cells, and data were analyzed by one-way ANOVA (Fisher’s LSD test with values p < 0.05 considered statistically significant, (*p <0.05, **p < 0.01, *** p < 0.001).

## Slide 2
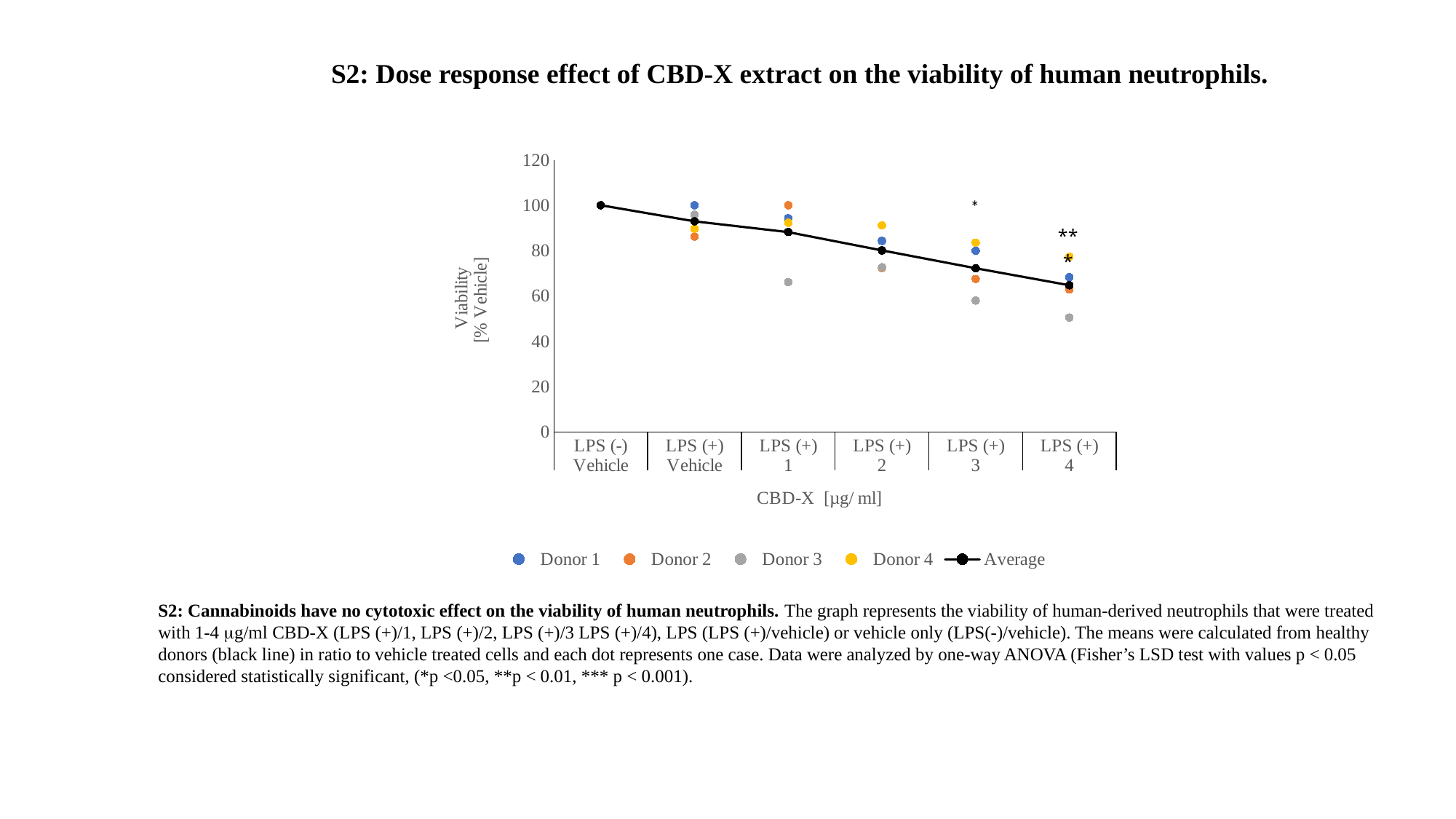

S2: Dose response effect of CBD-X extract on the viability of human neutrophils.
### Chart
| Category | Donor 1 | Donor 2 | Donor 3 | Donor 4 | Average |
|---|---|---|---|---|---|
| LPS (-) | 100.0 | 100.0 | 100.0 | 100.0 | 100.0 |
| LPS (+) | 100.0 | 86.15079692790486 | 95.80838323353294 | 89.64935004857288 | 92.90213255250268 |
| LPS (+) | 94.23647678314984 | 100.0 | 66.1018388754406 | 92.27922468427626 | 88.15438508571667 |
| LPS (+) | 84.26998563906174 | 72.31811049632505 | 72.63345649127277 | 91.09034556136373 | 80.07797454700582 |
| LPS (+) | 79.92340832934418 | 67.49525146585185 | 57.947933919395254 | 83.52916685941621 | 72.22394014350188 |
| LPS (+) | 68.2479655337482 | 62.826547746854956 | 50.45228691553064 | 77.25632603969098 | 64.69578155895618 |*
S2: Cannabinoids have no cytotoxic effect on the viability of human neutrophils. The graph represents the viability of human-derived neutrophils that were treated with 1-4 mg/ml CBD-X (LPS (+)/1, LPS (+)/2, LPS (+)/3 LPS (+)/4), LPS (LPS (+)/vehicle) or vehicle only (LPS(-)/vehicle). The means were calculated from healthy donors (black line) in ratio to vehicle treated cells and each dot represents one case. Data were analyzed by one-way ANOVA (Fisher’s LSD test with values p < 0.05 considered statistically significant, (*p <0.05, **p < 0.01, *** p < 0.001).

## Slide 3
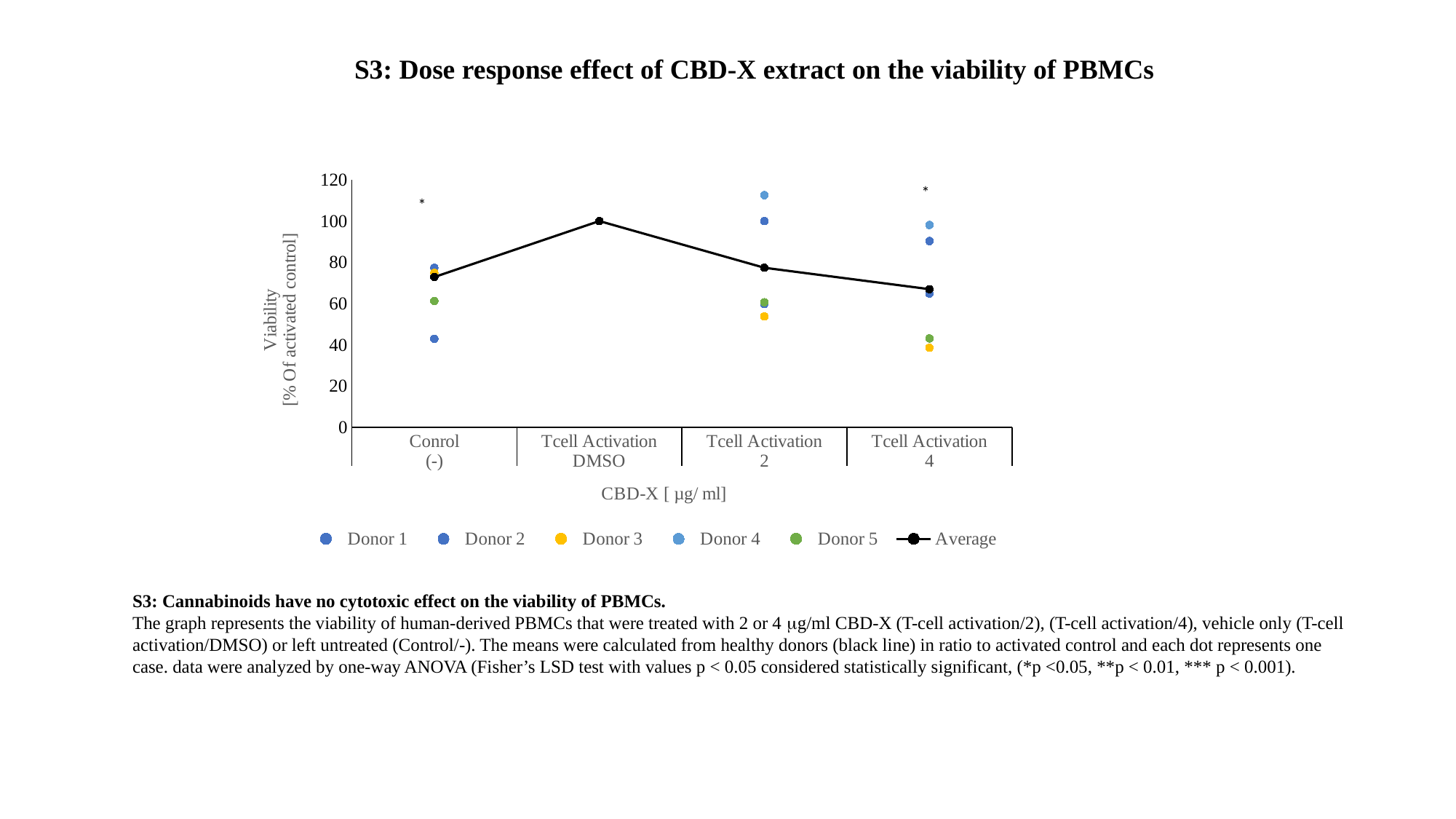

S3: Dose response effect of CBD-X extract on the viability of PBMCs
### Chart
| Category | Donor 1 | Donor 2 | Donor 3 | Donor 4 | Donor 5 | Average |
|---|---|---|---|---|---|---|
| Conrol | 77.31526239944544 | 42.90747138222412 | 74.82580891476567 | 107.95418049459373 | 61.2609956263207 | 72.85274376346993 |
| Tcell Activation | 100.0 | 100.0 | 100.0 | 100.0 | 100.0 | 100.0 |
| Tcell Activation | 59.803724849966244 | 100.0 | 53.78925264865897 | 112.56824751097312 | 60.62214359427982 | 77.35667372077562 |
| Tcell Activation | 64.8273471844731 | 90.31018146062394 | 38.54156724253126 | 98.11583342254576 | 43.04388422035481 | 66.96776270610579 |S3: Cannabinoids have no cytotoxic effect on the viability of PBMCs.
The graph represents the viability of human-derived PBMCs that were treated with 2 or 4 mg/ml CBD-X (T-cell activation/2), (T-cell activation/4), vehicle only (T-cell activation/DMSO) or left untreated (Control/-). The means were calculated from healthy donors (black line) in ratio to activated control and each dot represents one case. data were analyzed by one-way ANOVA (Fisher’s LSD test with values p < 0.05 considered statistically significant, (*p <0.05, **p < 0.01, *** p < 0.001).

## Slide 4
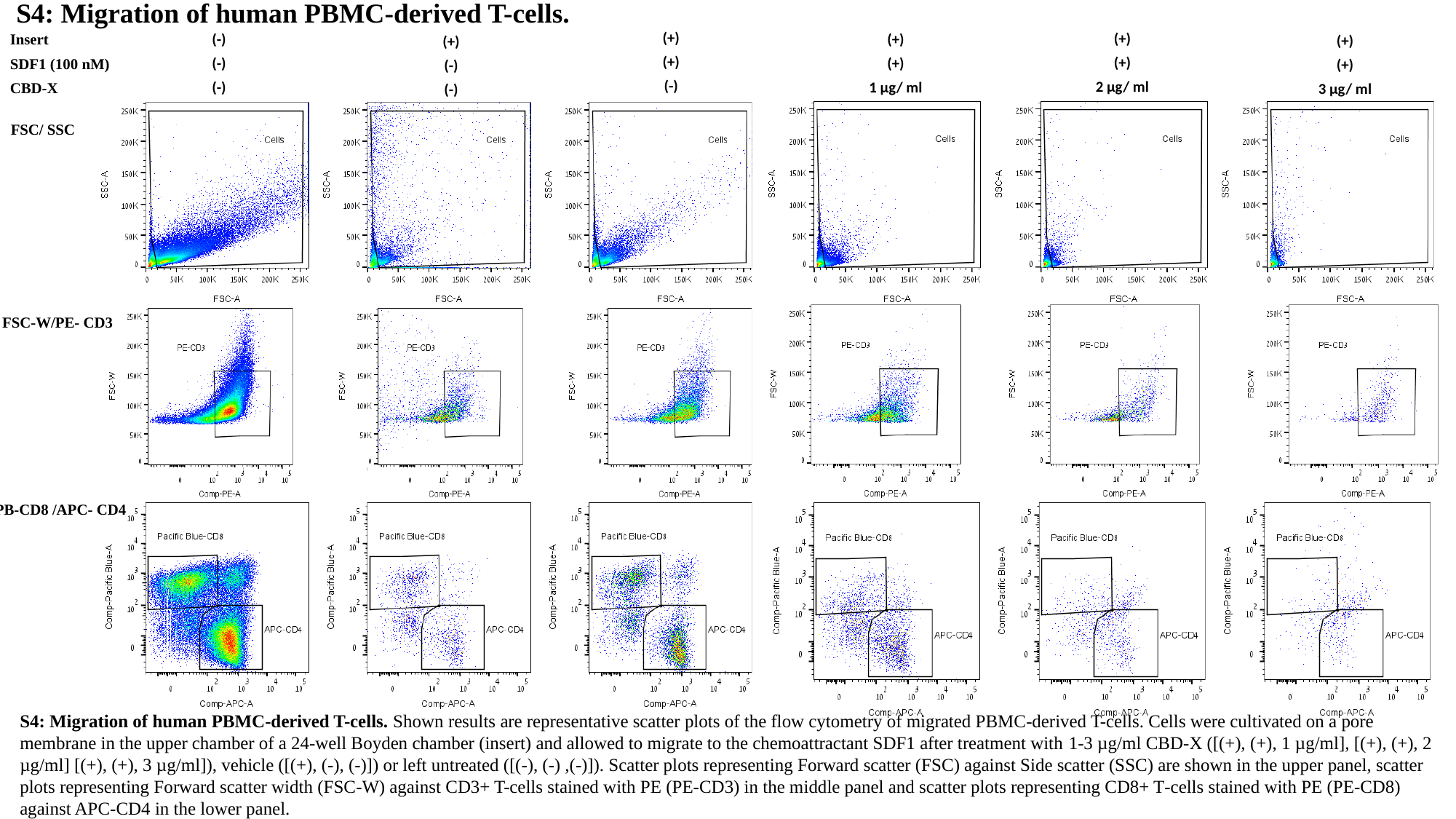

S4: Migration of human PBMC-derived T-cells.
| (+) |
| --- |
| (+) |
| (-) |
| (+) |
| --- |
| (+) |
| 2 µg/ ml |
| (-) |
| --- |
| (-) |
| (-) |
| (+) |
| --- |
| (+) |
| 1 µg/ ml |
| Insert |
| --- |
| SDF1 (100 nM) |
| CBD-X |
| (+) |
| --- |
| (+) |
| 3 µg/ ml |
| (+) |
| --- |
| (-) |
| (-) |
FSC/ SSC
FSC-W/PE- CD3
PB-CD8 /APC- CD4
S4: Migration of human PBMC-derived T-cells. Shown results are representative scatter plots of the flow cytometry of migrated PBMC-derived T-cells. Cells were cultivated on a pore membrane in the upper chamber of a 24-well Boyden chamber (insert) and allowed to migrate to the chemoattractant SDF1 after treatment with 1-3 µg/ml CBD-X ([(+), (+), 1 µg/ml], [(+), (+), 2 µg/ml] [(+), (+), 3 µg/ml]), vehicle ([(+), (-), (-)]) or left untreated ([(-), (-) ,(-)]). Scatter plots representing Forward scatter (FSC) against Side scatter (SSC) are shown in the upper panel, scatter plots representing Forward scatter width (FSC-W) against CD3+ T-cells stained with PE (PE-CD3) in the middle panel and scatter plots representing CD8+ T‑cells stained with PE (PE-CD8) against APC-CD4 in the lower panel.

## Slide 5
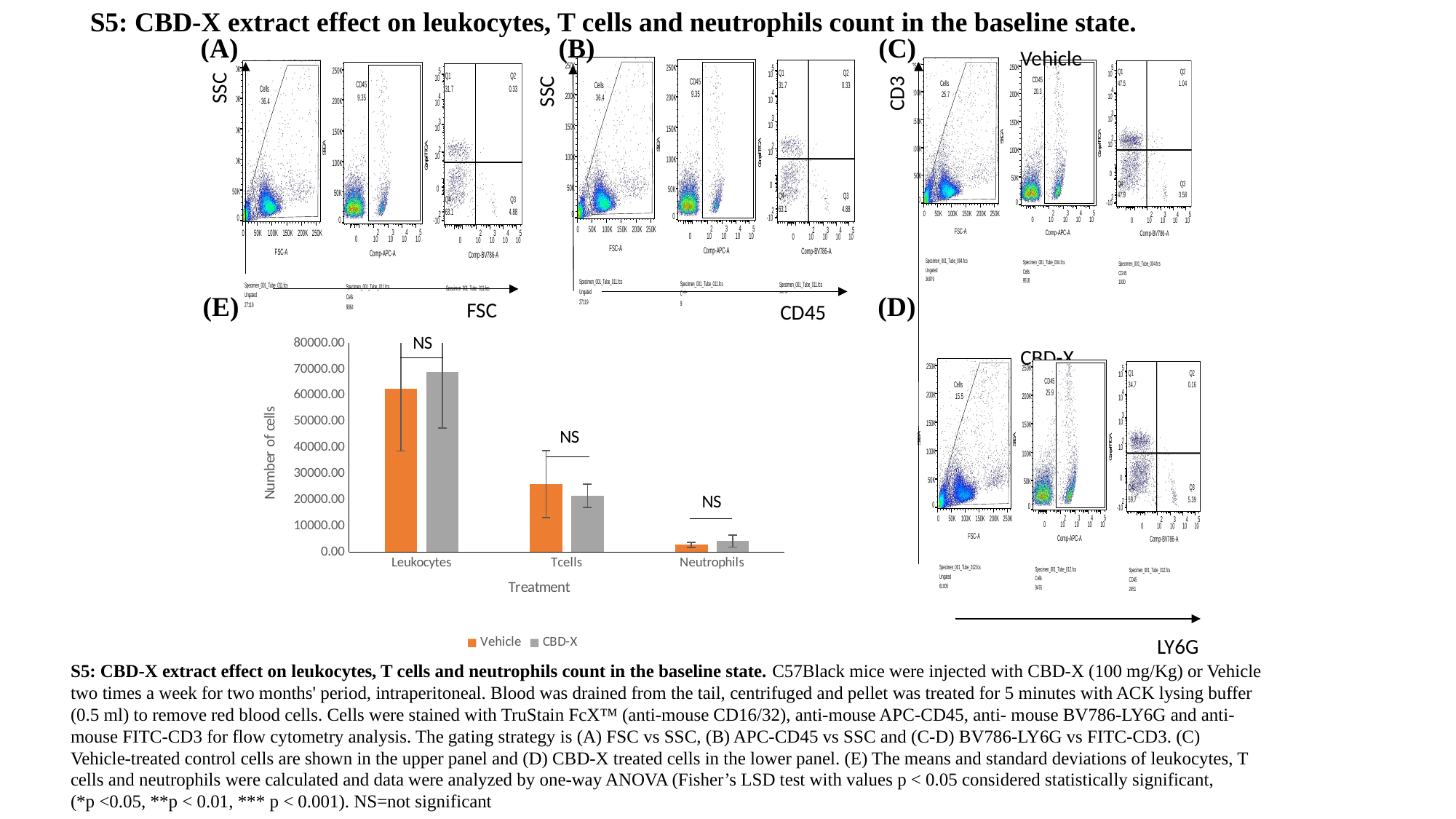

S5: CBD-X extract effect on leukocytes, T cells and neutrophils count in the baseline state.
(A)
(B)
(C)
Vehicle
SSC
CD3
SSC
(E)
(D)
FSC
CD45
### Chart
| Category | | |
|---|---|---|
| Leukocytes | 62471.220000000016 | 69098.79000000001 |
| Tcells | 25998.094860000005 | 21527.04675 |
| Neutrophils | 2739.1194120000005 | 4145.383569 |CBD-X
LY6G
S5: CBD-X extract effect on leukocytes, T cells and neutrophils count in the baseline state. C57Black mice were injected with CBD-X (100 mg/Kg) or Vehicle two times a week for two months' period, intraperitoneal. Blood was drained from the tail, centrifuged and pellet was treated for 5 minutes with ACK lysing buffer (0.5 ml) to remove red blood cells. Cells were stained with TruStain FcX™ (anti-mouse CD16/32), anti-mouse APC-CD45, anti- mouse BV786-LY6G and anti-mouse FITC-CD3 for flow cytometry analysis. The gating strategy is (A) FSC vs SSC, (B) APC-CD45 vs SSC and (C-D) BV786-LY6G vs FITC-CD3. (C) Vehicle-treated control cells are shown in the upper panel and (D) CBD-X treated cells in the lower panel. (E) The means and standard deviations of leukocytes, T cells and neutrophils were calculated and data were analyzed by one-way ANOVA (Fisher’s LSD test with values p < 0.05 considered statistically significant, (*p <0.05, **p < 0.01, *** p < 0.001). NS=not significant

## Slide 6
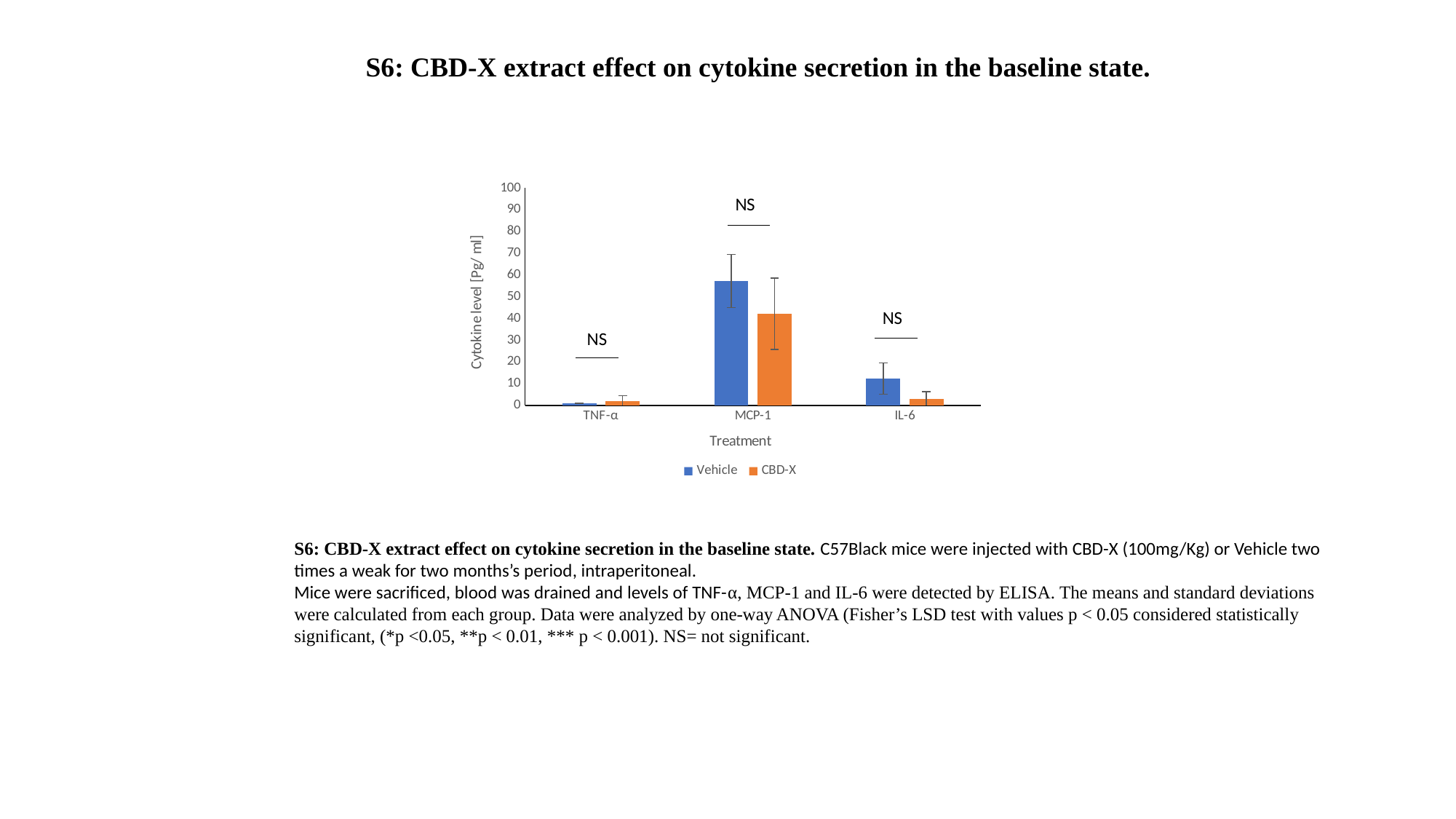

S6: CBD-X extract effect on cytokine secretion in the baseline state.
### Chart
| Category | Vehicle | CBD-X |
|---|---|---|
| TNF-α | 1.0 | 2.0 |
| MCP-1 | 57.242000000000004 | 42.0994 |
| IL-6 | 12.3744 | 2.99125 |NS
S6: CBD-X extract effect on cytokine secretion in the baseline state. C57Black mice were injected with CBD-X (100mg/Kg) or Vehicle two times a weak for two months’s period, intraperitoneal.
Mice were sacrificed, blood was drained and levels of TNF-α, MCP-1 and IL-6 were detected by ELISA. The means and standard deviations were calculated from each group. Data were analyzed by one-way ANOVA (Fisher’s LSD test with values p < 0.05 considered statistically significant, (*p <0.05, **p < 0.01, *** p < 0.001). NS= not significant.
